# Call for Ethical Guidelines in Cosmetic Dermatology for Adolescents

**DOI:** 10.1111/srt.70373

**Published:** 2026-08-19

**Authors:** Isabella J. Tan, Julia L. DiOrio, Bernard A. Cohen

**Affiliations:** ^1^ Rutgers Robert Wood Johnson Medical School New Brunswick New Jersey USA; ^2^ Department of Dermatology The Johns Hopkins Hospital Baltimore Maryland USA

**Keywords:** adolescent cosmetic procedures, collaboration, developmental appropriateness, ethical standards, holistic framework, medical necessity

## Abstract

**Background:**

The prevalence of appearance‐enhancing dermatology procedures among adolescents has increased markedly, raising ethical concerns regarding body image, developmental suitability, and psychosocial risk. Individuals 19 and under experienced a 75% increase in neuromodulator injections and a 71% increase in hyaluronic acid fillers between 2019 and 2022. Social and commercial forces—including social media and targeted marketing—promote cosmetic intervention as routine self‐optimization, despite adolescents’ ongoing cognitive, emotional, and identity development.

**Materials and Methods:**

We focus on elective, nonmedically necessary procedures such as neuromodulators and fillers in individuals aged 13–19. We review ethical principles (autonomy, beneficence, non‐maleficence, justice), psychosocial vulnerabilities (including body dysmorphic disorder [BDD]), social media influence, commercial pressures, and longitudinal mental health outcomes. Current regulatory guidance and professional society recommendations are assessed for gaps.

**Results:**

Adolescents’ emerging autonomy is developmentally incomplete, and appearance‐based motivations are often transient and externally influenced. Longitudinal data link cosmetic procedures with higher depression, anxiety, and lower self‐esteem. BDD affects ∼2% of adolescents, who rarely improve after intervention and may worsen. Social media exposure doubles the likelihood of considering cosmetic procedures, and commercial marketing further normalizes aesthetic ideals. Existing professional guidelines rarely mandate validated psychosocial screening, structured counseling, or cooling‐off periods, leaving adolescents vulnerable to premature intervention.

**Conclusion:**

A standardized, multi‐component ethical framework is necessary to guide clinicians caring for adolescents seeking elective aesthetic procedures. Recommendations include medical‐necessity assessment, structured counseling, strengthened parental consent, validated BDD screening, and careful documentation with follow‐up. Clear, evidence‐informed ethical standards are essential to safeguard adolescent well‐being and prioritize patient welfare over commercial interest.

## 1 Background

The prevalence of appearance‐enhancing (aesthetic) dermatology procedures among adolescents has increased markedly in recent years, prompting ethical concerns regarding body image, developmental suitability, and the potential psychosocial risks associated with such interventions. In individuals aged 19 and under, there was a 75% increase in neuromodulator injections and a 71% increase in hyaluronic acid (HA) fillers between the years 2019 and 2022 [1]. This paper defines adolescents as individuals aged 13–19 and focuses on elective, nonmedically necessary procedures such as neuromodulators and fillers. Social and commercial forces increasingly encourage adolescents to view cosmetic intervention as routine self‐optimization. Given adolescents’ ongoing cognitive, emotional, and identity development, these trends raise concerns about decision‐making capacity, susceptibility to external pressure, and long‐term psychological harm. Therefore, we argue for the adoption of a standardized, principle‐based ethical framework to guide clinicians, which we outline below.

## 2 Ethical Principles in Adolescent Care

Ethical decision‐making in adolescent cosmetic dermatology should be grounded in established bioethical principles: respect for autonomy, beneficence, non‐maleficence, and justice.

### 2.1 Autonomy

While adolescents possess emerging decisional capacity, autonomy in cosmetic decision‐making is developmentally incomplete. Appearance‐based motivations are often transient and heavily influenced by peers, parents, and social media. Clinicians must therefore assess not only assent, but decisional maturity, stability of motivation, and the degree of external influence. Autonomy alone is insufficient justification for elective cosmetic intervention in minors.

### 2.2 Beneficence and Non‐Maleficence

Clinicians are ethically obligated to act in the patient's best interest and avoid harm. Data demonstrating increased depression, anxiety, body dissatisfaction, and body dysmorphic disorder (BDD) exacerbation following adolescent cosmetic procedures directly implicate the principle of non‐maleficence. When the likelihood of psychological harm outweighs potential benefit—particularly in purely aesthetic interventions—procedural restraint is ethically required.

### 2.3 Justice

Access to cosmetic procedures is inconsistent and increasingly shaped by socioeconomic status, social capital, and digital influence. Normalizing aesthetic intervention in adolescence risks reinforcing appearance‐based inequities and shifting medical resources toward commercially driven care rather than health‐promoting interventions.

## 3 Influence of Social Media

Social media use has surged within adolescent age groups across platforms, shaping perceptions of beauty, with digitally altered images and celebrity culture setting unrealistic standards. Many images and videos online are altered to improve the looks of the individual captured, which can be difficult for an adolescent to recognize. Tethered images can contribute to distorted perceptions of normal and achievable beauty standards, which younger generations internalize for comparison. Celebrities further promote unrealistic expectations by denying cosmetic procedures and claiming natural beauty. A 2021 survey found that 72% of surgeons reported patients under 30 seeking procedures influenced by social media and selfies, up from 55% in 2015 [2]. Additionally, a study of 1,501 adolescents who used social media daily were twice as likely to consider a cosmetic procedure (OR = 2.05, *p < 0.001*) [3]. This appearance‐focused feedback loop underscores the necessity of routine psychosocial screening prior to any elective aesthetic intervention.

## 4 Commercial and Marketing Pressures

Beyond social media, direct‐to‐consumer marketing and the rapid expansion of medspas targeting younger clients have intensified demand. Promotional pricing, influencer partnerships, and aesthetic “maintenance” messaging normalize procedures traditionally reserved for adults. In these settings, commercial incentives may conflict with ethical clinical judgment, further underscoring the need for enforceable standards rather than discretionary practice norms.

## 5 Body Image and Psychosocial Maturity

The assessment of body image concerns is critical prior to undertaking cosmetic procedures in adolescents. Adolescents are navigating crucial phases of identity formation, making them especially vulnerable to body dissatisfaction. At this stage, an overemphasis on appearance can detract from the development of a healthy self‐image [4]. Cosmetic procedures may also worsen underlying psychological conditions, particularly in patients with BDD, which affects ∼2% of this population [5]. Importantly, adolescents with BDD rarely experience sustained improvement following cosmetic intervention, and symptoms may worsen.

Longitudinal data demonstrate that adolescents who undergo cosmetic procedures report higher depression and anxiety and lower self‐esteem up to five years post‐procedure [6, 7]. In a US‐based cohort study, adolescents receiving cosmetic procedures reported poorer mental health outcomes and greater dissatisfaction (*d* = 0.41, *p < 0.01*) compared with matched controls, even after adjusting for baseline distress [8].

Clinical evaluation must distinguish between motivations grounded in functional impairment versus those driven by peer comparison, bullying, parental pressure, or social media influence. For example, HA filler to correct congenital lip asymmetry with functional impact differs ethically from neuromodulator use for developmentally typical forehead lines in a 14‐year‐old [9, 10].

## 6 Current Regulations and Gaps

Although professional guidelines emphasize informed consent and parental involvement, few mandate validated psychosocial screening, structured counseling, or documentation of external pressure. Mental health assessment is rarely required, and cooling‐off periods are uncommon. This regulatory vacuum leaves adolescents vulnerable to premature and potentially harmful interventions.

## 7 Call to Action for Professional Societies and Proposed Solutions

We encourage the American Academy of Dermatology (AAD), in collaboration with the American Academy of Pediatrics (AAP) and the American Psychiatric Association (APA), to consider forming a joint task force to develop consensus guidance on elective aesthetic procedures in adolescents. Such guidance could recommend the use of validated psychosocial screening tools, structured consultation protocols that assess decisional maturity and potential external pressures, and consideration of psychological contraindications prior to intervention (Table 1). The task force might also provide suggestions for standardized documentation and follow‐up to support ongoing monitoring of mental health outcomes. By offering clear, evidence‐informed guidance rather than prescriptive mandates, professional societies can support clinicians in navigating complex ethical decisions while prioritizing adolescent well‐being.

**TABLE 1:** Proposed ethical guidelines for adolescent aesthetic dermatology.

Guideline component	Specific recommendations	Implementation/guidance examples
Evidence‐based assessment of medical necessity	Differentiate elective versus medically indicated procedures	Document the clinical rationale; for example, consider HA filler for congenital asymmetry with functional impact versus neuromodulators for mild physiologic lines in early adolescence. Guidance rather than strict cutoff is encouraged.
Counseling sessions	Structured interviews addressing motivation and expectations	Example questions: “What change do you hope this procedure will make in your daily life?” “How would you feel if you chose not to undergo this procedure?” Clinicians are encouraged to use these prompts to guide discussion rather than mandate responses.
Strengthening parental consent	Comprehensive discussions with adolescent and guardian	Consider a “cooling‐off” period (e.g., 2–4 weeks) between consultation and procedure to support informed decision‐making. Document discussions of risks, benefits, and external pressures.
Screening for BDD	Use validated tools to identify potential psychological risk	Tools such as BDD‐YBOCS or BIDQ may be used as part of the evaluation. High‐risk scores can prompt referral to a mental health professional, with clinician discretion guiding next steps.
Documentation and follow‐up	Record psychosocial rationale and outcomes	Maintain structured notes in the EHR including screening results, motivation assessment, and observed external pressures. Schedule follow‐up visits to review satisfaction and mental health, as clinically indicated.

## Funding

The authors have nothing to report.

## Conflicts of Interest

The authors declare no conflicts of interest.

## Data Availability

The data that support the findings of this study are available from the corresponding author upon reasonable request.
